# Lagovirus Non-structural Protein p23: A Putative Viroporin That Interacts With Heat Shock Proteins and Uses a Disulfide Bond for Dimerization

**DOI:** 10.3389/fmicb.2022.923256

**Published:** 2022-07-07

**Authors:** Elena Smertina, Adam J. Carroll, Joseph Boileau, Edward Emmott, Maria Jenckel, Harpreet Vohra, Vivien Rolland, Philip Hands, Junna Hayashi, Matthew J. Neave, Jian-Wei Liu, Robyn N. Hall, Tanja Strive, Michael Frese

**Affiliations:** ^1^Health and Biosecurity, Commonwealth Scientific and Industrial Research Organisation, Canberra, ACT, Australia; ^2^Faculty of Science and Technology, University of Canberra, Canberra, ACT, Australia; ^3^Centre for Invasive Species Solutions, Canberra, ACT, Australia; ^4^RSB/RSC Joint Mass Spectrometry Facility, Research School of Chemistry, Australian National University, Acton, ACT, Australia; ^5^Centre for Proteome Research, Department of Biochemistry & Systems Biology, Institute of Systems, Molecular and Integrative Biology, University of Liverpool, Liverpool, United Kingdom; ^6^Imaging and Cytometry Facility, John Curtin School of Medical Research, Acton, ACT, Australia; ^7^Agriculture and Food, Commonwealth Scientific and Industrial Research Organisation, Canberra, ACT, Australia; ^8^Research School of Chemistry, Australian National University, Acton, ACT, Australia; ^9^Australian Centre for Disease Preparedness, Commonwealth Scientific and Industrial Research Organisation, Geelong, VIC, Australia; ^10^Land and Water, Commonwealth Scientific and Industrial Research Organisation, Canberra, ACT, Australia

**Keywords:** *Caliciviridae*, SILAC, heat shock protein, viroporin, non-structural protein

## Abstract

The exact function(s) of the lagovirus non-structural protein p23 is unknown as robust cell culture systems for the *Rabbit haemorrhagic disease virus* (RHDV) and other lagoviruses have not been established. Instead, a range of *in vitro* and *in silico* models have been used to study p23, revealing that p23 oligomerizes, accumulates in the cytoplasm, and possesses a conserved C-terminal region with two amphipathic helices. Furthermore, the positional homologs of p23 in other caliciviruses have been shown to possess viroporin activity. Here, we report on the mechanistic details of p23 oligomerization. Site-directed mutagenesis revealed the importance of an N-terminal cysteine for dimerization. Furthermore, we identified cellular interactors of p23 using stable isotope labeling with amino acids in cell culture (SILAC)-based proteomics; heat shock proteins Hsp70 and 110 interact with p23 in transfected cells, suggesting that they ‘chaperone’ p23 proteins before their integration into cellular membranes. We investigated changes to the global transcriptome and proteome that occurred in infected rabbit liver tissue and observed changes to the misfolded protein response, calcium signaling, and the regulation of the endoplasmic reticulum (ER) network. Finally, flow cytometry studies indicate slightly elevated calcium concentrations in the cytoplasm of p23-transfected cells. Taken together, accumulating evidence suggests that p23 is a viroporin that might form calcium-conducting channels in the ER membranes.

## Introduction

**FIGURE 1:**
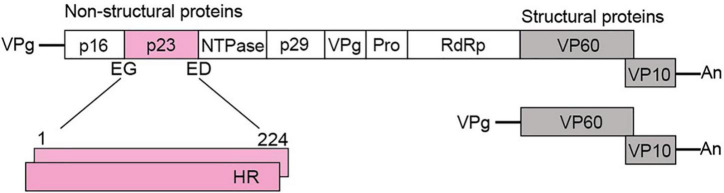
Schematic representation of a typical lagovirus genome. Both genomic **(Top)** and subgenomic RNAs **(Bottom)** contain two ORFs, are covalently linked to the viral protein VPg at the 5′-end, and are polyadenylated at the 3′-end (An). The genomic RNA encodes non-structural (shown in white and pink) and structural proteins (shown in gray), whereas the subgenomic RNA encodes only structural proteins. The coding sequence for p23 (224 amino acid residues) is shown in pink; two copies are shown to indicate that p23 dimerizes; cleavage sites at the N-terminus (E, glutamine and G, glycine) and C-terminus (E, glutamine and D, aspartic acid) are indicated; HR, hydrophobic region.

The *Rabbit haemorrhagic disease virus* (RHDV), genus *Lagovirus*, family *Caliciviridae*, infects only lagomorphs (i.e., rabbits, hares, and related leporids; the detailed classification and nomenclature of lagoviruses can be found in Supplementary Text 1). RHDV is of particular importance in Australia, as this virus is used as a biocontrol agent to manage the introduced European rabbit, one of the most devastating vertebrate pest species in the country (Kerr et al., 2021). Caliciviruses are small, non-enveloped viruses with a positive-sense, single-stranded RNA genome. Calicivirus particles contain two types of RNA molecules: a genomic RNA of about 7.5 kb (encodes all virus proteins) and a subgenomic RNA of about 2 kb that encodes only the capsid proteins (Ehresmann and Schaffer, 1977) (Figure 1). Genomic and subgenomic RNAs both contain two open reading frames (ORFs) (Meyers et al., 1991a,b). The first ORF of the genomic RNA encodes a polyprotein that is cleaved by the viral protease into seven non-structural (NS) proteins, namely p16 (NS1), p23 (NS2), helicase (NS3), p29 (NS4), VPg (NS5), protease (NS6), RNA-dependent RNA polymerase (RdRp; NS7), and the major structural protein VP60 (Meyers et al., 2000; Thumfart and Meyers, 2002). The second ORF encodes only the minor structural protein VP10. The subgenomic RNA is colinear with the 3′-end of the genomic RNA, i.e., it also encodes the two structural proteins (Boga et al., 1992; Morales et al., 2004). The main role of the structural proteins is to build the viral capsid while the non-structural proteins support viral replication and translation, counteract host immune responses, and possibly execute other functions (reviewed in Smertina et al., 2021).

Due to the lack of a robust cell culture system and reproducible reverse genetics system, rabbit caliciviruses remain poorly characterized. For example, the function(s) of the NS proteins p16, p23, and p29 have not yet been discovered. Nevertheless, recombinant proteins have been used to identify at least some features of these proteins. Here, we focus on p23 (originally named p23 after its apparent molecular weight, but later studies on the cleavage site between p23 and NS3 revealed that p23 is longer and has a molecular weight of approximately 25 kDa). The protein has an ER-like intracellular localization pattern and an ability to oligomerize (Urakova et al., 2015). The C-termini of p23 and homologous proteins from other caliciviruses are relatively conserved and hydrophobic, whereas the N-terminal part of these proteins is highly variable and largely disordered, i.e., it lacks secondary structure (Baker et al., 2012; Strtak et al., 2019). Using bioinformatic prediction tools, we previously identified two amphipathic membrane-spanning helices at the C-terminus of the p23 protein of RHDV (Smertina et al., 2021). Similar helices have been described for the p23 homologs of other caliciviruses (Baker et al., 2012; Strtak et al., 2019). Furthermore, the NS1/2 precursor of the norovirus NS2, the NS2 of *Feline calicivirus* (genus *Vesivirus*), and the NS1/2 of *Tulane virus* (genus *Recovirus*) localize to the ER (and/or Golgi membranes), and some of these proteins also oligomerize (reviewed in Smertina et al., 2021). These features led to the discovery of viroporin activity in cells expressing the NS1/2 of *Tulane virus* (Strtak et al., 2019). It is therefore tempting to speculate that the p23 protein of RHDV is also a viroporin that forms ion channels upon oligomerization.

Viroporins are small proteins that contain at least one membrane-spanning amphipathic helix (Nieva et al., 2012). As the formation of a functional membrane channel requires more than one transmembrane helix, small viroporins need to oligomerize. Viroporins often target intracellular membranes, especially those of the ER, thereby altering intracellular ion concentrations (Nieva et al., 2012). For example, the NS1/2 proteins of *Tulane virus* and some noroviruses cause a release of Ca^2+^ ions from the ER to the cytoplasm (Strtak et al., 2019). Many secreted proteins and cell surface membrane proteins (e.g., MHC proteins) acquire correct folding in the ER lumen, a process that depends on specific concentrations of Ca^2+^ and other ions. The elevated Ca^2+^ level in the ER (100 μM compared to 10–100 nM in the cytoplasm) is achieved by the sarco/endoplasmic reticulum Ca^2+^-ATPase, which pumps Ca^2+^ from the cytoplasm into the ER lumen. Depleted Ca^2+^ levels in the ER can trigger an unfolded protein response, as chaperones and folding enzymes depend on high Ca^2+^ concentrations (Kleizen and Braakman, 2004). On the other hand, increased levels of Ca^2+^ in the cytoplasm support many steps of the viral life cycle, from entry to replication, and the release of viral particles (Zhou et al., 2009).

In this study, we propose a mechanism of p23 oligomerization. We also identify heat shock proteins (Hsps) as cellular interaction partners of p23. Our results suggest that RHDV p23 is a viroporin that may form ion channels in ER membranes.

## Materials and Methods

### Cell Culture and Stable Isotope Labeling With Amino Acids in Cell Culture Labeling

Rabbit kidney (RK-13) cells (obtained from the European Collection of Cell Cultures, Porton Down, United Kingdom; 00021715) were cultured in Eagle’s minimum essential medium (MEM) supplemented with 10% fetal bovine serum (FBS; Sigma-Aldrich, St. Louis, MO, United States), 2 mM Glutamax (Gibco, Thermo Fisher Scientific, Waltham, MA, United States), 100 μg/ml of streptomycin (Gibco), and 100 units/ml of penicillin (Gibco). Cells were maintained in 5% CO_2_ at 37°C in humidified incubators. For SILAC labeling, cells were cultured in SILAC MEM (Thermo Fisher Scientific, Waltham, MA, United States), a depleted medium that lacks the two amino acids lysine and arginine. Before use, SILAC MEM was supplemented with either unlabeled (‘light’) L-arginine and L-lysine (Sigma-Aldrich) or isotope-labeled (‘heavy’) ^13^C_6_^15^N_2_ L-lysine (Lys8) and ^13^C_6_^15^N_4_ L-arginine (Arg10) (Sigma-Aldrich). To avoid unlabeled amino acid contamination, dialyzed FBS (10 kDa cut-off; Gibco, Thermo Fisher Scientific) was used (protocol adapted from Emmott and Goodfellow, 2014). For the same reason, a non-enzymatic cell dissociation solution (Sigma-Aldrich) was used to dislodge the cells for passaging (Sigma-Aldrich). Cells were cultured for at least seven passages in ‘heavy’ medium, and incorporation of heavy isotope-labeled amino acids was confirmed by mass spectrometry (MS) analysis. Cryopreserved aliquots of labeled cells were used in all subsequent experiments.

### Rabbit Liver Samples

RHDV2-infected and uninfected control rabbit liver samples [described in a previous study (Neave et al., 2018)] were used for label-free quantification (LFQ). Briefly, rabbits were either infected with RHDV2 (genotype GI.1bP-GI.2) or mock-infected with PBS. The animals were humanely killed 24 h post-infection, and 25% liver homogenates were prepared in RNA-later buffer and stored at −20°C. Samples from three RHDV2-infected animals (K375, K376, and K378) and three uninfected animals (K3, K14, and K12) were used in this study. Liver samples were clarified at 0.2 *g* for 5 min at 4°C, and 100 mg of solid precipitate was collected into tubes with glass beads. Liver homogenates were lysed in 300 μl of PBS (pH 7.5) with 150 mM NaCl, 0.5 mM EDTA, 0.5% Igepal CA-630 with protease inhibitors (Roche, Basel, Switzerland), 1 mM MgCl_2_, and 1 μl/ml benzonase nuclease (5 KU; Sigma-Aldrich). The suspension was homogenized using a Precellys homogenizer (Bertin Technologies, Montigny-le-Bretonneux, France) and incubated on ice for 30 min with gentle rocking to allow for full lysis. Finally, the lysates were cleared by centrifugation and supernatants were used for MS sample preparation.

### Plasmids and Transfections

Coding sequences for p23 (genotype GI.1cP) and green fluorescent protein (GFP) were cloned into the pCMV-Tag2C expression vector (Agilent Technologies, CA, United States) as described previously (Urakova et al., 2015) to generate N-terminally FLAG-tagged constructs (FLAG:p23 and FLAG:GFP). The Q5 site-directed mutagenesis kit (NEB) was used to generate constructs with cysteine to serine substitutions at the C-terminus of p23 (C451S, C456S, and a double substitution variant), at the N-terminus (C41S), a triple cysteine to serine variant, and p23 Δ146–224 truncated variant, as per manufacturer’s instructions. All constructs were verified by Sanger sequencing at the Biomolecular Resource Facility of The John Curtin School of Medical Research (Australian National University, Canberra, ACT, Australia). Lipofectamine 3000 (Invitrogen, Thermo Fisher Scientific) was used for transfections following the manufacturer’s guidelines. For SILAC experiments, antibiotic- and FBS-free SILAC medium was used in place of Opti-MEM (Gibco, Thermo Fisher Scientific). All SILAC experiments were performed in triplicate with one label-swap sample. For affinity purifications, labeled RK-13 cells were plated on 10-cm dishes and, when 70–80% confluent, transfected with 14 μg of plasmid complexed with Lipofectamine 3000. After transfection, cells were incubated overnight in 5% CO_2_ at 37°C prior to lysis and affinity purification.

### Cell Lysis and Affinity Purification

Transfected cells were washed with ice-cold PBS and scraped into a centrifuge tube with 1 ml PBS. Harvested cells were centrifuged at 200 *g*, resuspended in 400 μl of lysis buffer [PBS, pH 7.5 with 150 mM NaCl, 0.5 mM EDTA, 0.5% Igepal CA-630, and protease inhibitors (Roche)] and incubated on ice for 20 min with gentle rocking. Remaining cell debris was removed by centrifugation, and the supernatants were subjected to affinity purification after a 50-μl sample was set aside for Western blotting. Total protein concentration in the lysates was estimated with the bicinchoninic acid (BCA) protein assay (Abcam, Cambridge, United Kingdom) to ensure that the same amount of protein lysate was used for all affinity purifications. To reduce the concentration of Igepal CA-630, the lysates were diluted with an equal volume of washing buffer without Igepal CA-630 prior to affinity purification. After equilibration with washing buffer, anti-FLAG M2 affinity gel beads (25 μl per sample) (Sigma-Aldrich) were incubated with the cell lysates overnight at 4°C with rotation. After binding, the beads were washed three times with 500 μl of washing buffer. To elute the proteins from the beads, 60 μl of elution buffer [100 mM triethylammonium bicarbonate (TEAB), pH 7.55 with 5% (w/v) sodium dodecyl sulfate (SDS)] was added and samples were incubated for 10 min at 95°C. To collect the eluates, samples were centrifuged at 8,600 *g* for 2 min at room temperature and another aliquot was set aside for Western blotting. Eluates from p23 affinity purifications were combined with eluates from GFP affinity purifications at a 1:1 ratio (by volume), resulting in a total of three samples per experiment.

### Sample Preparation for Mass Spectrometry Analysis

#### Stable Isotope Labeling With Amino Acids in Cell Culture Samples

Combined eluates (a 1:1 mixture of p23 and GFP or 1:1 RdRp and GFP) were subjected to MS sample preparation using suspension trapping (s-trap; Protifi, Huntington, NY, United States) (Zougman et al., 2014), as per the manufacturer’s recommendations. Briefly, samples were reduced with 20 mM dithiothreitol and alkylated with 40 mM iodoacetomide (Sigma-Aldrich). S-trap micro columns were used for trypsin digestion; columns were incubated overnight at 37°C with 2.5 ng/μl of trypsin (Promega, Madison, WI, United States) per sample. Resulting peptides were diluted as recommended by the manufacturer and dried using a SpeedVac concentrator. Dried peptides were stored at −20°C before resuspension in 0.1% formic acid. Samples were filtered (0.2 μm; Millex, Merck Millipore, Darmstadt, Germany) prior to MS processing.

#### Global Proteome Analysis (Rabbit Liver Samples)

Liver lysates were subject to a BCA assay (Abcam), and a total of 100 μg of protein from each sample was loaded onto s-trap micro columns (Protifi), as described above. Trypsin digestion was performed overnight with 1.5 μl of trypsin (Promega) per sample. Eluted peptides were dried using a SpeedVac and fractionated with a High pH Reversed-Phase Peptide Fractionation kit (Pierce, Thermo Fisher Scientific), as recommended by the manufacturer. Briefly, the peptides were dissolved in 300 μl of 0.1% trifluoroacetic acid and loaded onto conditioned C18 columns (provided with the fractionation kit). After washing, the peptides were sequentially eluted with 5, 7.5, 10, 12.5, 15, 17.5, 20, and 50% acetonitrile in 0.1% triethylammonium bicarbonate. All fractions were dried and resuspended in 0.1% formic acid prior to MS processing.

### Western Blotting

Cell lysates were mixed with SDS-PAGE sample buffer (with or without β-mercaptoethanol to create reducing and non-reducing conditions, respectively), denatured by boiling for 5 min, and separated on 4–20% TruPAGE pre-cast gels (Sigma-Aldrich). Proteins were transferred onto a nitrocellulose transfer membrane (NitroBind, MSI, Cole-Parmer, IL, United States) using the Bio-Rad (Hercules, CA, United States) wet transfer blotting module. The membrane was subsequently blocked in 5% skim milk (Coles Supermarkets, Canberra, Australia) in tris-buffered saline with 0.1% Tween-20 (TBST) for 2 h at room temperature. Incubation with primary antibodies was performed overnight at 4°C in TBST. Incubation with secondary antibodies conjugated to horseradish peroxidase was performed for 1 h at room temperature in TBST. SIGMAFAST 3,3′-diaminobenzidine tablets (Sigma-Aldrich) were used for visualization of bands according to the manufacturer’s instructions. Staining of β-actin was used as a loading control where appropriate.

### Mass Spectrometry Data Acquisition and Analysis

For LFQ, the digested protein samples were analyzed using data-dependent MS/MS on a Thermo Orbitrap Fusion ETD mass spectrometer coupled to a Dionex UltiMate 3000 RSLCnano liquid chromatography (LC) system via a Nanospray Flex nano-ESI ion source (Thermo Fisher Scientific). Following injection by the autosampler, peptides were initially trapped onto a C18 PepMap100 μ-precolumn (5-μm particle size, 100-Å pore size; Thermo Fisher Scientific; Part No. 160454) with a 15 μl/min flow of 5% (v/v) acetonitrile in water coming from the loading pump of the LC, for 10 min, at which point a valve was switched to place the trap between the nano pump and the nano LC column to begin chromatographic separation. The peptides were separated on a C18 column (ReproSil-Pur 120 C18-AQ, 1.9 μm particle size; Dr. Maisch, Ammerbuch, Germany; Part No. r119.aq.0001) packed in-house at the Joint Mass Spectrometry Facility (Australian National University) (Richards et al., 2015). The nano LC mobile phase gradient was as follows: flow rate of 350 nL/min; solvent A = MilliQ Ultra-Pure water + 2% LC/MS-grade acetonitrile + 0.1% (v/v) Optima LC/MS-grade formic acid; solvent B = MilliQ Ultra-Pure Water + 80% Optima LC/MS-grade acetonitrile + 0.1% (v/v) formic acid; 5% B from 0 to 15 min; 12.5% B at 20 min; 40% B at 95 min; 80% B at 97 min, hold at 80% B until 109 min; 5% B at 110 min; hold at 5% B until 120 min. Mass spectrometer settings were as follows: Application Mode = Peptide; Positive mode ESI with 2.3 kV static spray voltage; Ion Transfer Tube Temperature = 305°C; Expected LC Peak Width = 30 s; Advanced Peak Determination = ON; Default Charge State = 2; Internal Mass Calibration = OFF; Survey Scans had Orbitrap Resolution = 1,20,000, Scan Range 375–1,500 m/z, RF Lens = 60%, Maximum Inject Time = 50 ms; Monoisotopic Peak Determination = Peptide; AGC Target = 4,00,000; Intensity Threshold = 5.0E3; Include Charge States 2–7; Include undetermined charge states = OFF; Dynamic Exclusion was set to exclude for 60 s after 1 selection event with a mass tolerance of ±10 ppm; Exclude Isotopes = ON; Perform dependent scan on single charge state per precursor only = OFF; Precursor Priority = Most Intense; MS2 scans where performed on the IonTrap detector with an isolation window of 1.6 m/z, Scan Range Mode = AUTO, Isolation Offset = OFF, Activation Type = CID, Collision Energy = 35%, Scan Rate = Rapid, Maximum Injection Time = 35 ms; Duty Cycle was limited to 3 s. The same acquisition parameters were used for the SILAC samples, except the spray voltage was set to 2.4 kV and the targeted Mass Difference filter was used with the following settings: number of precursors in the targeted group = 2, Mass List: Lys-8 and Arg-10, Partner Intensity Range Relative to the Most Intense Precursor (%) 5–100, Mass tolerance: ±10 ppm, perform subsequent scan on: most intense ion in the pair, charge state requirement: ions must be the same.

#### Mass Spectrometry Raw Data Analysis

MaxQuant software (version 1.6.10.43) was used for peptide identification and protein quantification (Cox and Mann, 2008). The Andromeda search engine (Cox et al., 2011) was used to search against the *Oryctolagus cuniculus* (European rabbit) UniProt FASTA database (downloaded on 21st August 2019) and a custom library of RHDV2 FASTA protein sequences based on the BlMt-1 GI.1bP-GI.2 virus (GenBank accession KT280060.1). Default search parameters were used, including up to two ‘missed trypsin cleavage sites,’ ‘oxidation of methionine,’ and ‘N-terminal acetylation’ as variable modifications, and ‘carbamidomethylation of cysteine’ as a fixed modification. The data were searched against a reverse database and peptide-spectrum match and protein false discovery rate was set to 0.01. LFQ (Cox et al., 2014) was used for whole proteome analysis. The ‘match between runs’ option was not applied. For SILAC raw data analysis, the ‘light’ and ‘heavy’ labels were specified in MaxQuant. Re-quantify and match between runs options were applied in this case.

### Statistical Analysis and Data Visualization

#### Proteomics

The whole proteome data were analyzed in R version 4.0.2 (R Core Team, 2020) using the packages DEP (Zhang et al., 2018), Enhanced Volcano (Blighe et al., 2020), TopGO (Alexa and Rahnenfuhrer, 2020), and GOplot (Walter et al., 2015). Multiple testing adjusted *p*-values were used with a significance cut-off of <0.05.

The SILAC data analysis was performed with the Perseus software (version 1.6.10.45; Tyanova et al., 2016). The following protein groups were filtered out: ‘only identified by site,’ ‘by reversed sequence,’ and ‘potential contaminants.’ Normalized heavy/light SILAC ratios were converted to a log scale (log_2_ SILAC ratio) and filtered for proteins identified in at least two out of three replicates. Significantly enriched proteins were identified using one sample *t*-test (compared to log_2_ ratio = 0, or no difference with internal ‘light’ GFP control) with an unadjusted *p*-value of <0.05.

#### Transcriptomics (RNA Sequencing)

RNA sequencing for transcriptome analysis was conducted previously (Neave et al., 2018) using the same liver homogenates that were used for the whole proteome analysis described here. Briefly, TopHat 2.1.1 (Trapnell et al., 2009) was used to map the sequenced reads to the rabbit genome assembly OryCun2.0 (GenBank assembly accession GCA_000003625.1). HTseq was used to count the reads for each gene (Anders et al., 2015). For statistical analysis and visualization, the R packages DESeq2 (Love et al., 2014), Enhanced Volcano (Blighe et al., 2020), and topGO (Alexa and Rahnenfuhrer, 2020) were used. The significance cut-off for differential gene expression was set to a multiple testing adjusted *p*-value < 0.01.

### Immunofluorescence and Confocal Microscopy

For the full list of antibodies used in this work, please refer to the Supplementary Table 1.

RK-13 cells grown on 8-chamber slides (Nunc Lab-Tek II, Thermo Fisher Scientific) were transfected as described above. After overnight incubation, cells were fixed with 4% paraformaldehyde in PBS for 15 min, permeabilized with 0.25% Triton X-100 in PBS for 10 min, and incubated for 90 min with blocking solution (5% bovine serum albumin; Sigma-Aldrich) in PBS. All incubations were performed at room temperature. Primary and secondary antibodies were diluted in 0.1% Tween-20 in PBS and were incubated overnight at 4°C and for 1 h at room temperature, respectively. Cell nuclei were stained with 4′,6-diamidino-2-phenylindole (DAPI) (Sigma-Aldrich). Finally, cover slips were mounted onto glass slides with Fluoroshield mounting medium (Sigma-Aldrich). Samples were imaged using a Leica SP8 (Leica Microsystems, Germany) laser-scanning confocal microscope equipped with a 40× objective and controlled with the Leica Application Suite X 3.5.1.18803 software (Leica Microsystems, Germany). Cells were imaged in three separate tracks. In the first track, samples were excited with 405 nm and DAPI signals were collected between 420 and 440 nm, together with transmitted light; in a second track, samples were excited with 499 nm and AF488 signals were collected between 510 and 540 nm; and in the third track, samples were excited with 555 nm and AF555 signals were collected between 563 and 630 nm. Cells were imaged using z-stacks, but quantitative colocalization analysis was performed on single planes only, using Leica software. Data were statistically analyzed in RStudio (R version 4.2.0; R Core Team, 2020) using one-way analysis of variance (ANOVA) followed by a Tukey’s honest significant difference test; graphs were produced with ggplot2 R package (Gómez-Rubio, 2017).

### Flow Cytometry: Ca^2+^ Flux Measurement

Fluo-4 acetyloxymethyl ester (Fluo-4 AM) (Thermo Fisher Scientific) 2 mM stock solution was prepared in anhydrous dimethyl sulfoxide (DMSO) (Sigma-Aldrich). RK-13 cells were grown on 60-mm dishes until 70–80% confluent and were transfected using lipofectamine 3000 as described above. After 14 h incubation, cells were harvested with trypsin, resuspended in FBS-free Dulbecco’s Modified Eagle Medium (DMEM) and counted using a hemocytometer. Cells were diluted to 1 × 10^6^ cells/ml with FBS-free DMEM and loaded with 1.5 μM final concentration of Fluo-4 AM (Thermo Fisher Scientific). Immediately after that, cell suspensions were vortexed and incubated for 30 min at 37°C while being protected from light. Dulbecco’s phosphate buffered saline (DPBS) supplemented with 2% FBS was used to wash excess Fluo-4 AM from cells. After centrifugation, cells were resuspended to 1 × 10^6^ cells/ml in 2% FBS DPBS. LSRII flow cytometer (BD Biosciences, Franklin Lakes, NJ, United States) was used for data acquisition. Data analysis was performed using FlowJo software version 10.8.1 (Tree Star, Ashland, OR, United States) and R (R Core Team, 2020). To pool the results of four independent experiments, the mean fluorescence intensities for each condition in each experiment were summed and each mean values were divided by the sums, resulting in normalization coefficients. Individual fluorescence intensities values were then multiplied by the respective coefficient. These data were then analyzed using the one-way Kruskal–Wallis test and pairwise comparisons were performed using the Wilcoxon rank sum test with multiple testing correction.

### *In silico* Predictions

The Predictor of Natural Disordered Regions (PONDR^1^) was used to analyze p23 protein sequences of RHDV (NC_001543.1), EBHSV (NC_002615.1), and RCV (MF598302.1). The PONDR output is a plot that indicates the strength of the prediction at each region with a cut-off value equal to 0.5 (Xue et al., 2010). The PSIPRED Protein Analysis Workbench server^2^ (Buchan and Jones, 2019) was used to analyze the secondary structure of p23 proteins, the potential presence of membrane helices was analyzed with the MEMSAT-SVM algorithm (Nugent and Jones, 2010, 2012). Membrane helix composition was studied using wheel diagrams generated with HeliQuest^3^ (Gautier et al., 2008).

### Lagovirus p23 Protein Sequence Alignment

All publicly available lagovirus p23 sequences (*n* = 2025; 6th August 2021) were downloaded from the GenBank nucleotide database based on the search term ‘lagovirus’. Three sequences were excluded (EU552531, UGVC01000001, and KY437668) because they were either not a lagovirus sequence or were provided as a circular sequence. A nucleotide alignment was generated from the remaining 2,022 sequences using MAFFTv7.450 in Geneious Prime 2021.1.1^4^ using default settings. The nucleotide alignment was truncated to the annotated p23/p26 coding sequence (672 nt) and partial p23 coding sequences were removed (leaving *n* = 567 sequences). The alignment was translated, and an amino acid sequence alignment (224 amino acids) was generated using MAFFTv7.450 with default amino acid settings.

## Results

### Oligomerization of p23 Requires a Disulfide Bond

**FIGURE 2:**
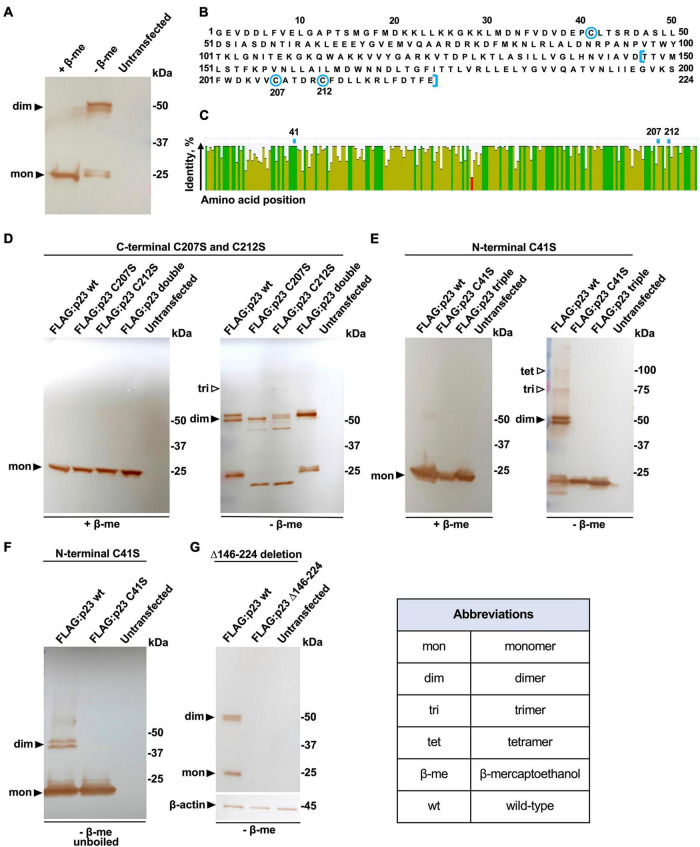
Dimerization of p23 depends on disulfide bond formation. **(A)** Recombinant FLAG-tagged p23 (wt, wild-type) expressed in transiently transfected RK-13 cells was eluted from an anti-FLAG resin with reducing or non-reducing Laemmli buffer containing β-mercaptoethanol (+β-me) or without β-mercaptoethanol (–β-me), respectively. Affinity purified p23 was separated by SDS-PAGE and analyzed by Western blotting using anti-FLAG antibodies; bands corresponding to monomeric p23 and dimeric forms can be observed. Untransfected RK-13 cell lysate was subject to immunoprecipitation as a negative control. The position of molecular mass standards is shown at the right. **(B)** RHDV/GI.1 p23 (NC_001543.1) protein sequence; the three cysteine residues at positions 41, 207, and 212 are highlighted using blue circles; and amino acids truncated in variant FLAG:p23 Δ146–224 are indicated using blue square brackets. **(C)** A consensus sequence and identity plot were generated using 567 lagovirus p23 sequences; the positions of the cysteine residues at position 41, 207, and 212 are indicated above the plot. The percentage identity at each amino acid position is indicated by column height and color (green means completely conserved, olive means less than complete identity, and red refers to a low identity). **(D)** Variants with C-terminal cysteine substitutions. Dimer formation of p23 wild-type (wt) and variants with cysteine-to-serine substitutions at positions 207 (C207S) or 212 (C212S) or both, ‘double’ was not affected under non-reducing conditions; the possible formation of a trimer is suggested by a weak band. **(E)** Dimers did not form between p23 variants with a cysteine-to-serine substitution at position 41 or in variants that had all cysteines substituted to serine (‘triple’); higher-order oligomers, trimers, and tetramers are seen in non-reducing conditions. **(F)** Unboiled samples were analyzed in a gel that did not contain SDS. Under these conditions, the C41S variant did not form dimers and both the wt protein and the C41S variant failed to form higher-order oligomers. In panel D, 10-μl samples were loaded per lane, and in E and F, 20-μl samples were loaded per lane (to enhance the detection of higher- order oligomers). **(G)** The truncated p23 Δ146–224 variant without transmembrane helices was not detected in either monomeric or dimeric form.

Previous experiments with recombinant proteins suggested that p23 forms dimers in transfected cells (Urakova et al., 2015). To better understand the molecular mechanism behind the oligomerization, we immunoprecipitated a N-terminally FLAG-tagged p23 (RHDV/GI.1) that was transiently expressed in rabbit kidney (RK-13) cells. Cell lysates were loaded on anti-FLAG antibody resin and proteins were eluted with either a reducing Laemmli buffer that contained β-mercaptoethanol or a non-reducing Laemmli buffer. Proteins in the eluate were separated and immunostained using SDS-PAGE and Western blotting. The experiment confirmed the previous observation that p23 forms dimers but also indicated that dimerization depends on the formation of one or more disulfide bonds, as the dimers were only observed in non-reducing conditions (Figure 2A). The protein sequence of p23 contains a total of three cysteine residues; one is located near the N-terminus (position 41) and two are located near the C-terminus (positions 207 and 212) (Figure 2B). All three cysteines are highly conserved, i.e., we found that all available lagovirus sequences contained cysteines at positions 41 and 212 and that nearly all contained a cysteine at position 207 (Figure 2C). These findings prompted us to investigate the role of cysteine residues in the oligomerization of p23 and check whether oligomerization depends on one or two intermolecular disulfide bonds. To find out whether one or more of these cysteines are indeed essential for oligomerization, site-directed mutagenesis was used to substitute cysteine with serine residues. Initially, we focused on the C-terminal cysteine residues and substituted: (*i*) the cysteine at position 207 (C207S), (*ii*) cysteine at position 212 (C212S), (*iii*) the cysteines at positions 207 and 212 (C207S/C212S, referred to as ‘double’). These protein variants were expressed and immunoprecipitated as described above; eluates were collected using reducing or non-reducing loading buffers, and samples were analyzed by Western blotting (Figure 2D). The C207S and C212S variants migrated noticeably faster under non-reducing conditions, which may be indicative of an intramolecular disulfide bond between the neighboring cysteine residues at positions 207 and 212. However, both variants were still able to dimerize, indicating that neither of the C-terminal cysteine residues is involved in dimerization. The result further suggests that the N-terminal cysteine at position 41 is likely involved in the formation of an intermolecular disulfide bond that stabilizes dimers. To test this hypothesis, we generated two additional variants by substituting (*iv*) the cysteine at position 41 (C41S) and (*v*) the cysteines at positions 41, 207, and 212 (C41S/C207S/C212S; referred to as ‘triple’). Our results clearly show that the N-terminal cysteine at position 41 is essential for the dimerization of p23 or for the stabilization of dimers, as no dimer was observed under non-reducing conditions for any of the variants that contain a serine at position 41 (Figure 2E). Moreover, additional bands indicate the presence of higher-order oligomers (trimers and tetramers) under non-reducing conditions (Figure 2E). In order to test whether hydrophobic interactions aid in the stabilization of dimers (and perhaps higher-order oligomers), some samples were processed under non-reducing conditions and without boiling. Under these conditions, we did not detect any oligomerization (Figure 2F), suggesting that the formation of the disulfide bond between cysteines at position 41 is the main driver behind the dimerization of p23. Finally, a truncated p23 variant was generated that retains the cysteine at position 41 but lacks 78 C-terminal amino acids (FLAG:p23 Δ146–224). According to earlier *in silico* predictions (Smertina et al., 2021), such a truncation will remove two transmembrane helices, and it would be interesting to determine whether this protein variant can still oligomerize. However, we were not able to detect FLAG:p23 Δ146–224 in transiently transfected cells (Figure 2G), suggesting that the variant is unstable and rapidly degraded.

### Lagovirus Protein p23 Contains Two Amphipathic Transmembrane Helices

**FIGURE 3:**
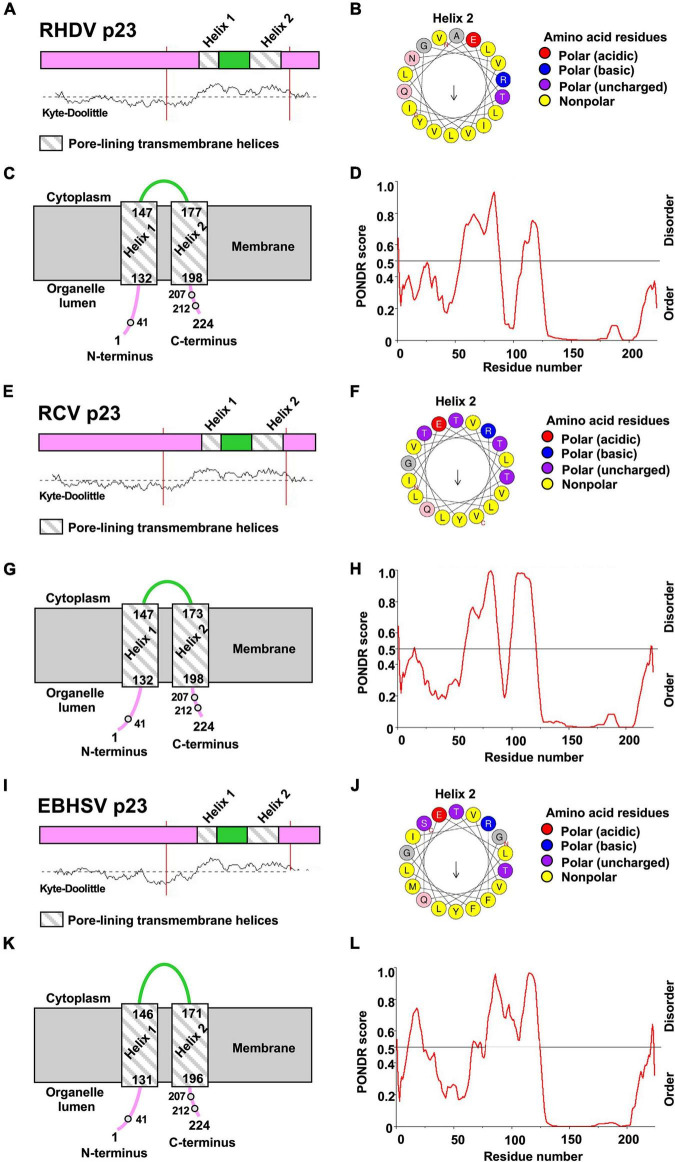
Prediction of transmembrane helices and disordered regions using *in silico* sequence analyses. **(A–D)** p23 protein sequences from *Rabbit haemorrhagic disease virus* (RHDV/GI.1; NC_001543.1), **(E–H)** *Rabbit calicivirus* (RCV/GI.4; MF598302.1), and **(I–L)** *European brown hare syndrome virus* (EBHSV/GII.1; NC_002615.1). **(A,C,E,G,I,K)** PSIPRED secondary structure prediction with MEMSAT-SVM algorithm and Kyte-Doolittle hydropathy plot revealed the presence of two putative transmembrane helices in the p23 proteins of all lagoviruses examined. The helices (‘helix 1’ and ‘helix 2’) are shown as striped boxes. **(D,H,L)** PONDR plot shows predicted disordered and ordered regions; the strength of the prediction is indicated by the score on the *y*-axis (>0.5 are considered disordered). **(B,F,J)** HeliQuest was used to generate a wheel diagram for the amino acid sequence of ‘helix 2.’ Arrows represent direction and magnitude of the hydrophobic moment. Note that the orientation of p23 within the membrane is not clear.

To better understand the mechanism and the role of p23 dimerization, we explored the secondary structure of p23 proteins from RHDV, *Rabbit calicivirus* (RCV), and *European brown hare syndrome virus* (EBHSV) using the predictor of natural disordered regions (PONDR) server (Xue et al., 2010) and the PSIPRED protein analysis workbench (Buchan and Jones, 2019). Most of the N-terminal 125 residues of all three proteins are hydrophilic and disordered, while the C-terminal region is hydrophobic and highly ordered (see Kyte-Doolittle and PONDR plots in Figure 3). Furthermore, the MEMSAT-SVM secondary structure prediction algorithm identified two pore-lining (amphipathic) helices near the C-terminus in all three lagovirus p23 proteins (Figures 3A,C,E,G,I,K), suggesting that all p23 proteins interact with membranes and possibly form a transmembrane channel. To further study the composition of these helices, HeliQuest (Gautier et al., 2008) was used to generate ‘helix wheels.’ The first helix (‘Helix 1,’ aa 132–147) appears too short to build a wheel. Therefore, only wheels for the second helix (‘Helix 2,’ aa 177–198) are shown (Figures 3B,F,J). In all cases, the results show a clear separation into mostly polar (hydrophilic) and non-polar (hydrophobic) regions, suggesting a conserved amphipathic nature of the predicted helices.

According to the secondary structure prediction, the cysteine at position 41 is further away from the membrane than cysteines 207 and 212, and is thus more accessible for a disulfide bond formation. The membrane orientation of the N- and C-termini is unknown; however, it is likely that both are located in the organelle lumen, i.e., the ER as this organelle provides a favorable oxidative environment for disulfide bond formation (Tu and Weissman, 2004). The observed oligomerization may lead to the formation of a transmembrane channel built from two or more p23 monomers.

### Recombinant p23 Localizes With the Endoplasmic Reticulum in Transfected Cells

**FIGURE 4:**
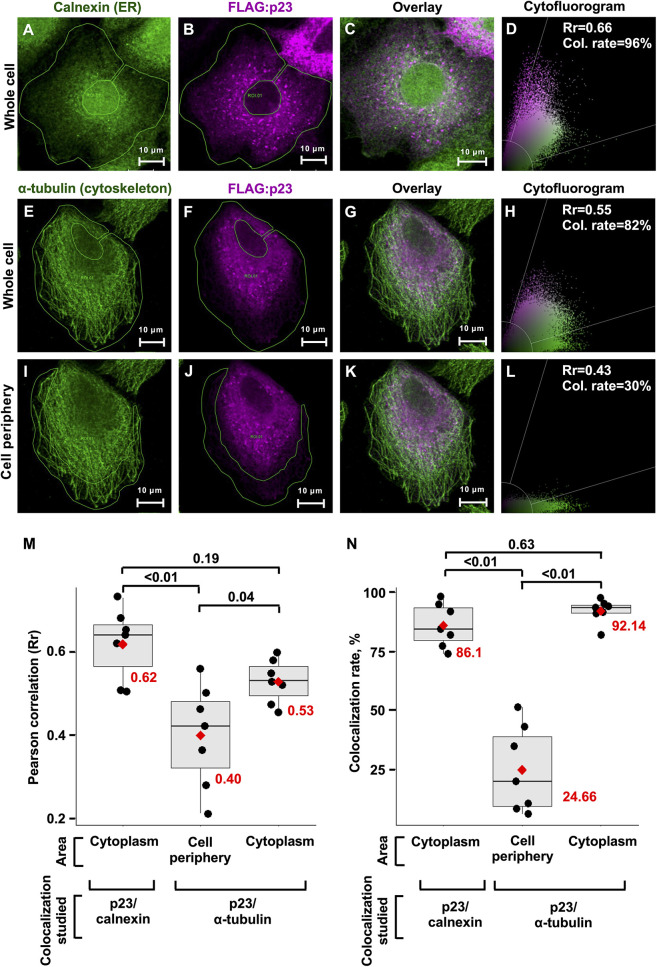
Transiently expressed recombinant p23 localizes to the ER. RK-13 cells were transfected with FLAG-tagged p23 (RHDV), incubated overnight, fixed, immunostained, and analyzed by confocal microscopy. **(A–C)** The ER marker calnexin (green) and FLAG-tagged p23 (magenta) were detected by immunofluorescence using anti-calnexin and anti-FLAG antibodies, respectively. **(E–H)** The cytoskeleton protein α-tubulin (green) and FLAG-tagged p23 (magenta) were detected by immunofluorescence using anti-tubulin and anti-FLAG antibodies, respectively. **(I–L)** The cell periphery lacks p23 expression and therefore this area was used to estimate the threshold for Rr. **(D,H,L)** The cytofluorograms show the distribution of green and magenta pixels in the cytoplasm (areas under investigation are bounded by a green line); Rr, Pearson correlation coefficient and Col. rate (%), colocalization rate. **(M,N)** Boxplots represent the distribution of Rr and colocalization rates for seven individual cells (*n* = 7), respectively. Each cell is depicted as a black dot, the median for each group is shown as a black line inside the boxes, the position of the mean is shown as a red diamond, and the value of mean is given in red numbers. Significance was analyzed using one-way ANOVA followed by Tukey’s honest significant difference test; adjusted *p*-values are shown above the plots; plots were produced using ggplot2 (Gómez-Rubio, 2017).

The p23 proteins of RHDV and RCV show an ER-like localization in transfected RK-13 cells (Urakova et al., 2015). Homolog proteins of many other caliciviruses were also found to colocalize with ER membranes, with only the p23 homolog of human norovirus (NS1/2) colocalizing with the Golgi (Ettayebi and Hardy, 2003; reviewed in Smertina et al., 2021). Our hypothesis that the p23 protein of lagoviruses is a viroporin prompted us to look more closely at the intracellular localization of p23. RK-13 cells were seeded in chamber slides and transfected to transiently express a FLAG-tagged p23. The p23 protein was co-stained either with the ER marker calnexin or α-tubulin, a major structural component of the cytoskeleton (Figures 4A–C,E–G). We found that p23 strongly colocalizes with calnexin in areas that contain ER membranes. In contrast, p23 does not always colocalize with α-tubulin, although p23 and α-tubulin staining often overlaps in the cytoplasm around the nucleus, the colocalization did not extend to the periphery where we detected strong α-tubulin signals but only weak p23 staining (Figures 4I–K). Furthermore, we quantified the colocalization of p23 and calnexin (Figures 4D,H,L). We determined the mean Pearson correlation coefficient (0.62) and the mean colocalization rate (86.10%) for 7 cells (*n* = 7) (Figures 4M,N). These data further support the earlier observation that these proteins colocalize. We also analyzed the potential colocalization of p23 and α-tubulin, for which we calculated a mean Pearson correlation coefficient and colocalization rate of 0.53 and 92.14%, respectively (*n* = 7). However, when we restricted our analysis to the periphery of cells, we found a mean Pearson correlation coefficient and colocalization rate of 0.40 and 24.66%, respectively (*n* = 7), suggesting that areas devoid of ER membranes do not contain much p23, likely because the majority of p23 is membrane associated. Nevertheless, it should be noted that p23 is translated by free ribosomes, which means that every p23 protein spends time in the cytoplasm, which would explain the weak staining in the periphery of cells and the observed Pearson correlation coefficient and colocalization rate with α-tubulin. Taken together, our results confirm a colocalization of p23 with the ER.

### p23 Interacts With Heat Shock Proteins

**FIGURE 5:**
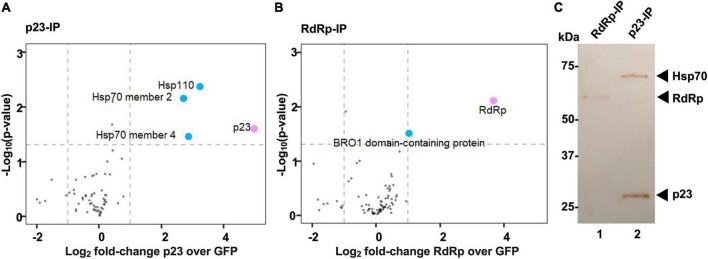
Heat shock proteins interact with p23 but not the viral polymerase. **(A,B)** A quantitative proteomics approach (SILAC) was used to identify cellular interaction partners of p23 and the RNA-dependent RNA polymerase (RdRp) of RHDV, respectively. Proteins identified in at least two out of three biological replicates were included in the analysis. Cellular proteins significantly enriched in comparison to an internal GFP control are shown as blue dots; the viral bait proteins p23 and RdRp are highlighted in pink. Significance was analyzed using a one-sample *t*-test with an unadjusted *p*-value of <0.05 and a log_2_ fold-change > 1. Heat shock proteins Hsp70 and Hsp90 were identified as p23 interactors **(A)**, and one or more BRO1 domain-containing proteins were identified as RdRp interactors **(B)**. **(C)** Western blot analysis of immunoprecipitated complexes isolated from transfected cells expressing recombinant FLAG:p23 or FLAG:RdRp (p23-IP and RdRp-IP, respectively). Aliquots of affinity purified complexes were separated by SDS-PAGE and analyzed by Western blot using anti-FLAG and anti-Hsp70 antibodies. Hsp70 was detected in p23 but not in RdRp expressing cells (we did not attempt to detect Hsp110 or BRO1 domain-containing proteins).

To identify cellular interaction partners of p23, we used stable isotope labeling of amino acids in cell culture (SILAC) coupled with immunoprecipitation. In this approach, a population of cells was grown for several passages in culture medium that was supplemented with heavy isotope-labeled arginine and lysine while control cells were cultured in unlabeled medium that contained regular arginine and lysine. Heavy isotope-labeled (‘heavy’) and unlabeled (‘light’) RK-13 cells were transiently transfected to express recombinant FLAG-tagged p23 and GFP [for heavy to light (heavy/light) ratio quantification], respectively. As a labeling control, transfections were swapped in one out of three replicates (i.e., GFP and p23 was expressed in ‘heavy’ and ‘light’ cells, respectively). Using quantitative MS to analyze the composition of affinity-purified FLAG-tagged protein complexes, we found that p23 specifically interacts with heat shock proteins, namely Hsp70 member 2, Hsp70 member 4, and Hsp110 (Figure 5A). To investigate the specificity of the observed interaction between p23 and heat shock proteins, another SILAC experiment was conducted with the viral RNA-dependent RNA-polymerase (RdRp) as bait (Figure 5B). In this case, no interaction with heat shock proteins was detected. Instead, we identified a BRO1 domain-containing protein as a potential interactor of RdRp. Western blotting analysis of the individual eluate aliquots from anti-FLAG resin (before p23 and GFP samples were combined) further demonstrated that p23 specifically interacts with heat shock proteins, as a band corresponding to Hsp70 was only detected when p23 was used as bait (Figure 5C).

### Global Transcriptome and Proteome Characterization of RHDV2-Infected Liver Samples Confirm Alterations in Ca^2+^ Homeostasis

**FIGURE 6:**
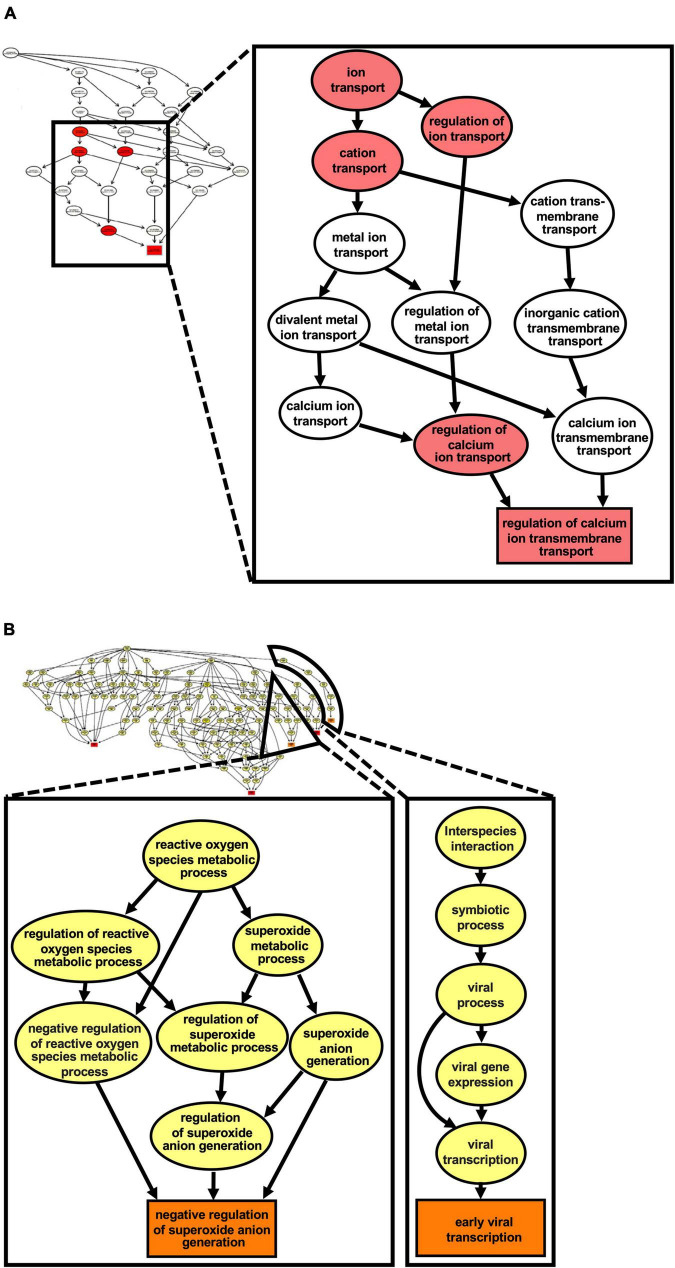
Ca^2+^ transmembrane transport pathways are altered in the liver of RHDV2- infected rabbits. A gene ontology (GO) enrichment analysis was conducted using the list of differentially expressed genes from RNA sequencing. **(A)** The graph induced by the top 5 GO terms identified by the weight01 algorithm (Alexa et al., 2006); the square shape indicates the most significant GO term. **(B)** The graph induced by the top 5 GO terms identified by weight01 algorithm with Fisher exact test also identified pathways associated with viral infection and regulation of reactive oxygen species generation. Box colors represents the relative significance of GO terms, from white (least significant) to red (most significant).

Due to the lack of a suitable cell culture system for RHDV, we investigated infected rabbit liver samples to identify cellular pathways that change during infection. We used RHDV2-infected rabbit liver tissue samples and data from a previous study (Neave et al., 2018) to characterize global changes to the transcriptome and proteome, respectively. The transcriptome analysis was performed using raw RNA sequencing data (‘reads’) as made available by Neave et al. (2018) (BioProject accession No. PRJNA434149). For the proteome analysis, three existing RHDV2-infected and three mock-infected liver samples that had been stored in RNA-later were processed for MS-based proteomic analysis. Differences in gene expression (either at the mRNA or protein level) between infected and uninfected samples were identified and visualized using volcano plots (Supplementary Figure 1); full lists of differentially expressed genes and proteins can be found in Supplementary Data Files 1, 2, respectively. Next, we performed a gene set enrichment analysis using topGO (Alexa and Rahnenfuhrer, 2020) to identify biological pathways and molecular functions of differentially expressed genes. This analysis yielded gene ontology (GO) terms that are associated with pathways such as fatty acid transport and metabolism, and cytoskeleton reorganization; pathways associated with unfolded protein and heat shock protein responses were also identified, although only at the transcript level (Table 1). We generated a ‘graphical output’ that shows the GO terms in the context of a greater network of pathways; a large amount of data was produced on numerous pathways of which only a small subset is displayed in Figure 6 (for full-size figures, see Supplementary Figures 2, 3).

**TABLE 1:** Gene ontology analysis of differentially expressed genes and proteins in RHDV2-infected rabbit liver samples.

GO term*	Description	*P*-value
**Transcriptome**		

**Biological pathway**		
0046719	Regulation by virus of viral protein levels in host cell	0.003
0090150	Establishment of protein localization to membrane	0.027
1903371	Regulation of ER tubular network organization	0.028
1903076	Regulation of protein localization to plasma membrane	0.033
**Molecular function**		
0003777	Microtubule motor activity	0.0001
0051082	Unfolded protein binding	0.0002
0004698	Calcium-dependent protein kinase C activity	0.004
0042803	Protein homodimerization activity	0.005
0030544	Hsp70 protein binding	0.020

**Proteome**		

**Biological pathway**		
1902001	Fatty acid transmembrane transport	0.006
2000191	Intracellular lipid transport	0.021
0031532	Actin cytoskeleton reorganization	0.039
2001234	Negative regulation of apoptotic signaling pathway	0.044
**Molecular function**		
0042803	Protein homodimerization activity	0.003
0043495	Protein-membrane adaptor activity	0.008
0005324	Long-chain fatty acid transporter activity	0.008
0000146	Microfilament motor activity	0.010
0051015	Actin filament binding	0.011

*Gene ontology terms (only selected terms are shown).

This analysis revealed changes to the Ca^2+^ homeostasis, which further supports our hypothesis that lagoviruses encode a viroporin that forms channels in intracellular membranes.

### Recombinant p23 Expression in Cultured Cells Slightly Changes Cytoplasmic Ca^2+^ Level

**FIGURE 7:**
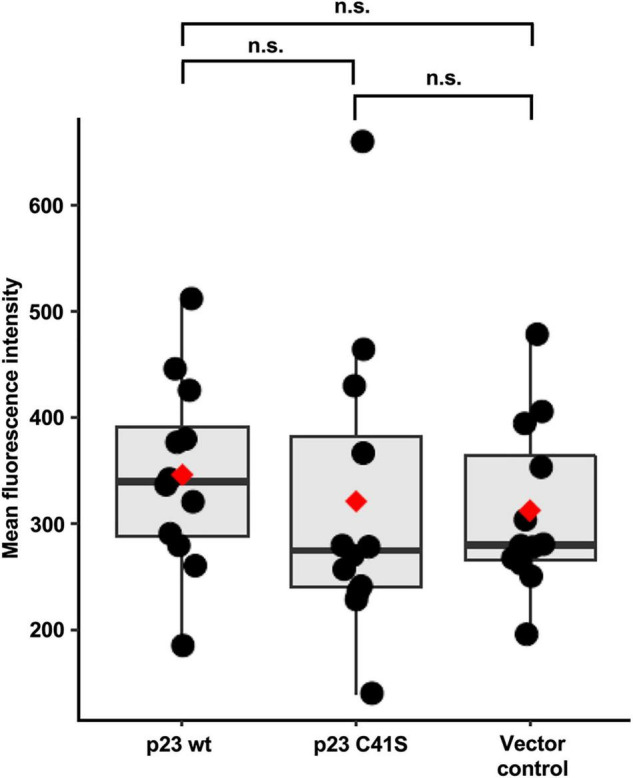
Flow cytometry Ca^2+^ flux measurements. Mean fluorescence intensity was measured for 25,000 cells in each condition, with three replicates per condition. Boxplots represent the distribution of mean fluorescence intensity value. Median values are shown as a horizontal line and mean values as a red diamond. The results from four independent experiments were normalized and combined. Significance was analyzed using one-way Kruskal-Wallis test and pairwise Wilcoxon rank test with multiple testing correction; adjusted *p*-values are shown above the plot; the plot was produced using ggplot2 (Gómez-Rubio, 2017); n.s., not significant (*p* > 0.05).

RK-13 cells were transiently transfected with plasmids encoding either RHDV FLAG:p23 or a mutated FLAG:p23 with the C41S substitution, or the plasmid vector (pCMV-Tag2C). After transfection, the cells were loaded with the cell-permeable fluorescent dye Fluo-4 AM (that increases fluorescence intensity upon Ca^2+^ binding by more than 100-fold) and the mean fluorescence intensity was measured with a flow cytometer. We observed that the mean fluorescence intensity in the cytoplasm increased slightly in cells expressing wild-type p23 compared to cells expressing the C41S variant or cells that were transfected with the ‘empty’ vector (Figure 7). However, the pooled results of four independent experiments showed that these differences were not statistically significant.

## Discussion

Our knowledge about many key aspects of the lagovirus life cycle is limited. For example, the function(s) of three out of seven non-structural proteins are still unknown. Closing this research gap is difficult as many caliciviruses, including lagoviruses, do not grow in cell culture. Therefore, we and others mostly relied on the expression of recombinant proteins to study their intracellular localization, enzymatic activity, and oligomerization. Furthermore, various screening and multi-omics approaches have been used to identify cellular interaction partners and characterize the effects of NS proteins on cellular metabolism. For example, Hosmillo et al. (2019) used proteomics to describe the ‘interaction network’ for the norovirus protein NS1/2 (NS2 is a positional homolog of p23). In that study, NS1/2 was shown to interact with the stress granule component G3BP1 and regulate cellular protein translation (Hosmillo et al., 2019). Furthermore, global transcriptome characterizations revealed that the transient expression of norovirus NS1/2 in transfected cells and during genuine norovirus infection leads to changes in Ca^2+^ homeostasis; more specifically, Lateef and co-authors found evidence for the downregulation of the Ca^2+^ voltage-gated channel subunit alpha 1D (Lateef et al., 2017).

Here, we focused on the p23 proteins of lagoviruses; our data suggest that these proteins are viroporins that reside in ER membranes, form dimers (and possibly also higher-order oligomers), and may act as ion channels. Furthermore, we found that p23 dimers are stabilized by an intermolecular disulfide bond between the cysteine residues at position 41. This disulfide bond is likely formed in the lumen of the ER, but further structural studies are warranted to confirm this hypothesis. The presence of faint bands in some of our Western blots suggests that p23 also forms trimers and tetramers, but the mechanism behind the formation of these higher-order oligomers and their potential biological relevance remains unknown.

Secondary structure prediction tools suggested that p23 positions itself into a membrane using two amphipathic helices in the C-terminal region of the protein. Oligomerization is a common mechanism in the formation of membrane channels, especially for small proteins. Indeed, viroporins are usually small proteins that require oligomerization. For example, the hepatitis C viroporin protein p7 is only 63 amino acids long and requires six monomers to build a transmembrane channel (Luik et al., 2009). The p23 homolog in *Tulane virus* (family *Caliciviridae*, genus *Recovirus*) is 233 amino acids long and is also a viroporin that disrupts Ca^2 +^ homeostasis by forming channels in the ER membranes after dimerization (Strtak et al., 2019). In noroviruses, the p23 homolog NS1/2 has a distinctly ordered hydrophobic C-terminal region, similar to that observed in lagoviruses and *Tulane virus* (Baker et al., 2012). A viroporin that also relies on disulfide bonds for oligomerization was recently described for *Feline calicivirus* (genus *Vesivirus*) (Peñaflor-Téllez et al., 2022). Moreover, the viroporins of *Feline calicivirus* (Barrera-Vázquez et al., 2019) and *Murine norovirus* (Robinson et al., 2019) trigger apoptosis, which may be a critical factor in efficient virus spreading. Accumulating evidence suggests that viroporin activity may be a feature that is conserved across the family *Caliciviridae* (reviewed in Smertina et al., 2021).

To confirm the hypothesis that p23 interacts with cellular membranes, we extended earlier confocal microscopy studies (Urakova et al., 2015) by conducting a quantitative colocalization analysis. We observed that RHDV p23 localizes with the ER marker calnexin in transfected cells, a finding that confirms previous results (Urakova et al., 2015). However, the detected colocalization was not complete, and multiple p23-specific puncta were located beyond the cytofluorogram colocalization threshold. This observation suggests that when p23 is overexpressed in cells, it may only be partially transported to and incorporated in the membranes.

A quantitative proteomics approach (SILAC) coupled with immunoprecipitation identified several heat shock proteins as potential interactors of p23. Heat shock proteins are known as common contaminants in proteomics studies (Trinkle-Mulcahy et al., 2008). However, we believe that the observed interaction is specific and has biological relevance as the same experiment was performed for another viral protein, the RNA-dependent RNA polymerase, and for this protein, we did not observe the interaction with heat shock proteins. It is tempting to speculate that the interaction between p23 and heat shock proteins is important to stabilize the partially hydrophobic p23 proteins in the cytoplasm and ‘guide’ them toward cellular membranes. Many viroporins, including p23, do not have a signal sequence and thus do not trigger the cellular signal recognition system that co-translationally directs proteins to the ER, therefore requiring alternative mechanisms of transportation. In several cases, cellular chaperones have been observed to assist such proteins post-translationally (Rabu et al., 2008; Martinez-Gil and Mingarro, 2015). Furthermore, cytosolic Hsp70 and co-chaperones are responsible for keeping tail-anchored membrane proteins soluble, and assist their transportation to the ER membranes in yeast cells (Cho and Shan, 2018). This function is likely also used by mammalian cells, as an association between heat shock proteins and newly synthesized membrane proteins was also detected in rabbit reticulocyte lysates (Abell et al., 2007; Colombo et al., 2009). In the case of viral infections, cellular chaperones switch their attention as protein quality assurers to viral proteins. Yellow fever virus polyprotein processing depends on Hsp40, a cochaperone that assists Hsp70-mediated protein folding (Bozzacco et al., 2016). Our observation that the p23 protein of RHDV interacts with heat shock proteins may therefore indicate that p23 requires additional stabilization prior to its oligomerization and interaction with cellular membranes.

Another point of discussion is whether the disulfide bonds between p23 monomers are formed in the ER or in the cytoplasm. It is known that the oxidative environment in the ER lumen promotes formation of disulfide bonds (Tu and Weissman, 2004); therefore, it is conceivable that monomeric p23 is transported to the ER where it interacts with the membranes in a manner that drives the N-terminus into the lumen of the organelle. Then, it becomes available for S-S bond formation with another p23. Moreover, we observed that p23 variants with C-terminal cysteine-to-serine substitutions show an altered SDS-PAGE mobility, which is indicative of an intramolecular disulfide bond. Thus, p23 can be classified as a class IIA viroporin as both its N- and C-termini are located in the ER lumen, similar to the hepatitis C virus protein p7 (Martinez-Gil and Mingarro, 2015). The fact that the transmembrane helices are located at the C-terminus and the cysteine residue responsible for the oligomerization at the N-terminus, raises a question whether a ‘classic’ viroporin domain (as defined in Hyser and Estes, 2015) can be defined for p23.

Most of our work has been conducted using transiently transfected rabbit kidney cells. To corroborate our findings, we performed global proteome and transcriptome analysis of infected rabbit liver samples. The differentially expressed proteins and genes were used to identify pathways that changed during the infection. Pathways associated with Ca^2 +^ signaling and unfolded protein response were among those identified by our analyses, which suggests that p23 may affect the Ca^2+^ homeostasis.

Finally, we conducted flow cytometry-based Ca^2+^ flux measurements in transiently transfected cells. These experiments did not reveal statistically significant differences between the analyzed groups (i.e., wild-type p23, C41S p23, and negative control). However, we observed a trend across four independent experiments pointing to slightly elevated Ca^2+^ levels in cells that expressed wild-type p23. Considering that the transfection efficiency was well below 100%, our experiments likely underestimated the effect of p23 expression on Ca^2+^ homeostasis. Furthermore, changes to intracellular Ca^2+^ levels are notoriously difficult to measure using fluorescent dyes (Perry et al., 2015), which means that additional studies are required to fully characterize the transport characteristics of ion channels in lagovirus replication.

Overall, our results extend the definition of a viroporin domain. These findings will benefit future work aimed at defining the 3D structure of p23 oligomers, an important step toward the development of new antiviral drugs.

## Data Availability Statement

The RNAseq dataset has been deposited in the NCBI Sequence Read Archive under the BioProject accession No. PRJNA434149. Proteomics data (label-free quantification) have been deposited to the ProteomeXchange Consortium via the PRIDE repository (Vizcaíno et al., 2016) with the dataset identifier PXD031027. The SILAC dataset has been deposited to the ProteomeXchange Consortium via the PRIDE repository with identifier PXD031619.

## Ethics Statement

Ethical review and approval was not required, because for this study, we used frozen liver samples from a previous study [Neave et al., 2018, Viruses, 10(9), 512; https://doi.org/10.3390/v10090512]. All previous animal experiments were conducted at the Commonwealth Scientific and Industrial Research Organisation (CSIRO) Black Mountain Laboratories following the Australian Code for the Care and Use of Animals for Scientific Purposes (2013) and approved by the CSIRO Ecosystem Sciences Animal Ethics Committee (permit identifiers: CESAEC DOMRAB, SEAEC 10-12, ESAEC 13-10).

## Author Contributions

ES, RH, TS, and MF conceptualized the project. RH, TS, and MF secured funding. ES, AC, JB, EE, MJ, HV, VR, PH, JH, J-WL, MN, RH, and MF acquired and analyzed the data. ES, JB, and MF wrote the first draft. All authors contributed to manuscript revision, read, and approved the submitted version.

## Conflict of Interest

The authors declare that the research was conducted in the absence of any commercial or financial relationships that could be construed as a potential conflict of interest.

## Publisher’s Note

All claims expressed in this article are solely those of the authors and do not necessarily represent those of their affiliated organizations, or those of the publisher, the editors and the reviewers. Any product that may be evaluated in this article, or claim that may be made by its manufacturer, is not guaranteed or endorsed by the publisher.

## Footnotes

1
http://www.pondr.com/

2
http://bioinf.cs.ucl.ac.uk/psipred/

3
https://heliquest.ipmc.cnrs.fr/

4
https://www.geneious.com
