# Supplementary material for: Lagovirus Non-structural Protein p23: A Putative Viroporin That Interacts With Heat Shock Proteins and Uses a Disulfide Bond for Dimerization
Source: Front Microbiol. 2022 Jul 7;13:923256. doi: 10.3389/fmicb.2022.923256 (PMC9340658; doi:10.3389/fmicb.2022.923256)
Supplement: Supplementary file 1 [file Image_1.PDF]

Supplementary Figure S1

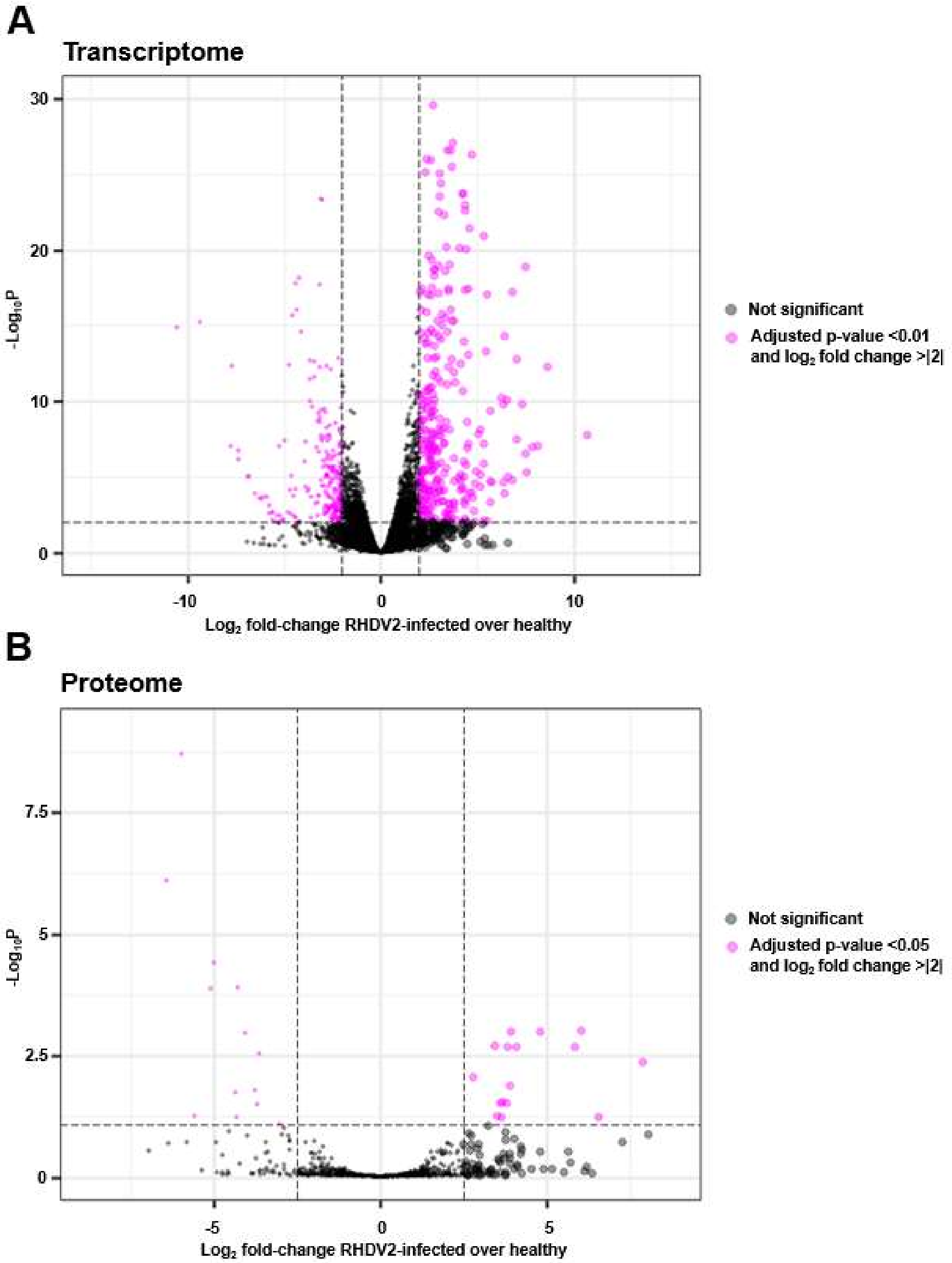

Differentially expressed genes in RHDV-infected livers. Volcano plots representing differentially expressed genes (**A**) and proteins (**B**) in liver samples from RHDV2-infected (24 hpi) and uninfected rabbits. Genes and proteins that were significantly upregulated or downregulated are depicted as large magenta dots, respectively. Non-significant gene and protein expression changes are shown in black. Dots are transparent to more easily distinguish overlapping points. The thresholds are derived at log_2 fold change > 2 or < -2 and significance was determined using adjusted p-values of <0.01 (transcriptome) and <0.05 (proteome). Full lists of differentially expressed genes and proteins can be found in **Supplementary Data File S1** and **Supplementary Data File S2**, respectively.
