# Supplementary material for: Lagovirus Non-structural Protein p23: A Putative Viroporin That Interacts With Heat Shock Proteins and Uses a Disulfide Bond for Dimerization
Source: Front Microbiol. 2022 Jul 7;13:923256. doi: 10.3389/fmicb.2022.923256 (PMC9340658; doi:10.3389/fmicb.2022.923256)
Supplement: Supplementary file 2 [file Table_1.docx]

Supplementary Table S1

List of antibodies

| **Antibody** | **Product number** | **Supplier** | **Dilution** | **Conjugation** |
| --- | --- | --- | --- | --- |
| **Primary antibodies** | | | | |
| Rat monoclonal anti-FLAG | NOVNBP106712SS | Novus Biologicals | 1:1500 (WB*)  1:150 (IF**) | Unconjugated |
| Rabbit monoclonal anti-Hsp70 | ab45133 | Abcam | 1:1500 (WB) | Unconjugated |
| Mouse monoclonal anti-β-actin | ab8226 | Abcam | 1:1500 (WB) | Unconjugated |
| Rabbit polyclonal anti-calnexin | ab75801 | Abcam | 1:100 (IF) | Unconjugated |
| Rabbit polyclonal anti-α-tubulin | PA5-19489 | Thermo Scientific | 1:100 (IF) | Unconjugated |
| **Secondary antibodies** | | | | |
| Secondary goat anti-rabbit | ab150077 | Abcam | 1:300 (IF) | Alexa Fluor 488 |
| Secondary goat anti-rat | ab150158 | Abcam | 1:300 (IF) | Alexa Fluor 555 |
| Secondary goat anti-rat | 3050-05 | Cambridge Bioscience | 1:3000 (WB) | HRP*** |
| Secondary goat anti-mouse | 170-6516 | Bio-Rad | 1:3000 (WB) | HRP |
| Secondary goat anti-rabbit | 170-6515 | Bio-Rad | 1:3000 (WB) | HRP |

* Western blotting; ** immunofluorescence; *** horseradish peroxidase
