# Supplementary material for: Lagovirus Non-structural Protein p23: A Putative Viroporin That Interacts With Heat Shock Proteins and Uses a Disulfide Bond for Dimerization
Source: Front Microbiol. 2022 Jul 7;13:923256. doi: 10.3389/fmicb.2022.923256 (PMC9340658; doi:10.3389/fmicb.2022.923256)
Supplement: Supplementary file 3 [file Data_Sheet_1.DOCX]

Supplementary Text S1

Lagoviruses are hierarchically classified based on the phylogeny of the major structural protein VP60 (Le Pendu et al., 2017). The classification system consists of genogroups (GI, GII), genotypes (e.g., GI.1, GI.2, GI.3, etc.), and variants (e.g., GI.1a, GI.1b, GI.1c, etc.). Genogroup II lagoviruses include benign enterotropic viruses and highly virulent hepatotropic viruses that typically infect hares (*Lepus* species) (Droillard et al., 2020). Genogroup I comprises several genotypes; those that are specific to rabbits such as the virulent and hepatotropic GI.1 viruses (Liu et al., 1984) and the benign enterotropic GI.3 (Le Gall-Reculé et al., 2011) and GI.4 (Strive et al., 2009) viruses; as well as GI.2 viruses (also known as RHDV2), which infect rabbits (*Oryctolagus*), hares (*Lepus*), jackrabbits (*Lepus*), and cottontails (*Sylvilagus*) (Lankton et al., 2021).

Caliciviruses frequently undergo recombination at the RdRp-VP60 junction, combining non-structural and structural genes from different genotypes and even different genogroups (Mahar et al., 2021; Szillat et al., 2020). Recombinant viruses are named Gx.xP-Gx.x, where P denotes the polymerase (or non-structural) variant. A characterisation of recombinant viruses by Mahar et al (2021) revealed that the determinants of tissue tropism and virulence are associated with the structural gene coding regions. Antigenicity and evasion of humoral immune responses are also conferred by the VP60 major capsid protein (Wang et al., 2013). However, the non-structural proteins are critical for viral replication and can also confer epidemiological fitness advantages independent of the capsid protein (Mahar et al., 2021).

**References**

Droillard, C., Lemaitre, E., Chatel, M., Quéméner, A., Briand, F. X., Guitton, J. S., et al. (2020). Genetic diversity and evolution of Hare Calicivirus (HaCV), a recently identified lagovirus from Lepus europaeus. *Infect. Genet. Evol.* 82, 104310. doi:10.1016/j.meegid.2020.104310.

Lankton, J. S., Knowles, S., Keller, S., Shearn-Bochsler, V. I., and Ip, H. S. (2021). Pathology of Lagovirus europaeus GI.2/RHDV2/b (Rabbit Hemorrhagic Disease Virus 2) in Native North American Lagomorphs. *J. wildl. dis* 57, 694–700. doi:10.7589/JWD-D-20-00207.

Le Gall-Recul, G., Zwingelstein, F., Boucher, S., Le Normand, B., Plassiart, G., Portejoie, Y., et al. (2011). Virology: Detection of a new variant of rabbit haemorrhagic disease virus in France. *Vet. Rec.* 168, 137–138. doi:10.1136/vr.d697.

Le Pendu, J., Abrantes, J., Bertagnoli, S., Guitton, J. S., Le Gall-Reculé, G., Lopes, A. M., et al. (2017). Proposal for a unified classification system and nomenclature of lagoviruses. *J. Gen. Virol.* 98, 1658–1666. doi:10.1099/jgv.0.000840.

Liu, S. J., Xue, H. P., Pu, B. Q., and Qian, N. H. (1984). A new viral disease in rabbits. *Anim. Husb. Vet. Med. (Xumu yu Shouyi)* 16, 253–255.

Mahar, J. E., Jenckel, M., Huang, N., Smertina, E., Holmes, E. C., Strive, T., et al. (2021). Frequent intergenotypic recombination between the non-structural and structural genes is a major driver of epidemiological fitness in caliciviruses. *Virus Evol.* 7, 1–14. doi:10.1093/ve/veab080.

Strive, T., Wright, J. D., and Robinson, A. J. (2009). Identification and partial characterisation of a new lagovirus in Australian wild rabbits. *Virology* 384, 97–105. doi:10.1016/j.virol.2008.11.004.

Szillat, K. P., Hoper, D., Beer, M., and Konig, P. (2020). Full-genome sequencing of German rabbit haemorrhagic disease virus uncovers recombination between RHDV (GI.2) and EBHSV (GII.1). *Virus Evol.* 6, 1–11. doi:10.1093/ve/veaa080.

Wang, X., Xu, F., Liu, J., Gao, B., Liu, Y., Zhai, Y., et al. (2013). Atomic Model of Rabbit Hemorrhagic Disease Virus by Cryo-Electron Microscopy and Crystallography. *PLoS Pathog.* 9. doi:10.1371/journal.ppat.1003132.
