# Supplementary material for: Lagovirus Non-structural Protein p23: A Putative Viroporin That Interacts With Heat Shock Proteins and Uses a Disulfide Bond for Dimerization
Source: Front Microbiol. 2022 Jul 7;13:923256. doi: 10.3389/fmicb.2022.923256 (PMC9340658; doi:10.3389/fmicb.2022.923256)
Supplement: Supplementary file 6 [file Data_Sheet_4.PDF]

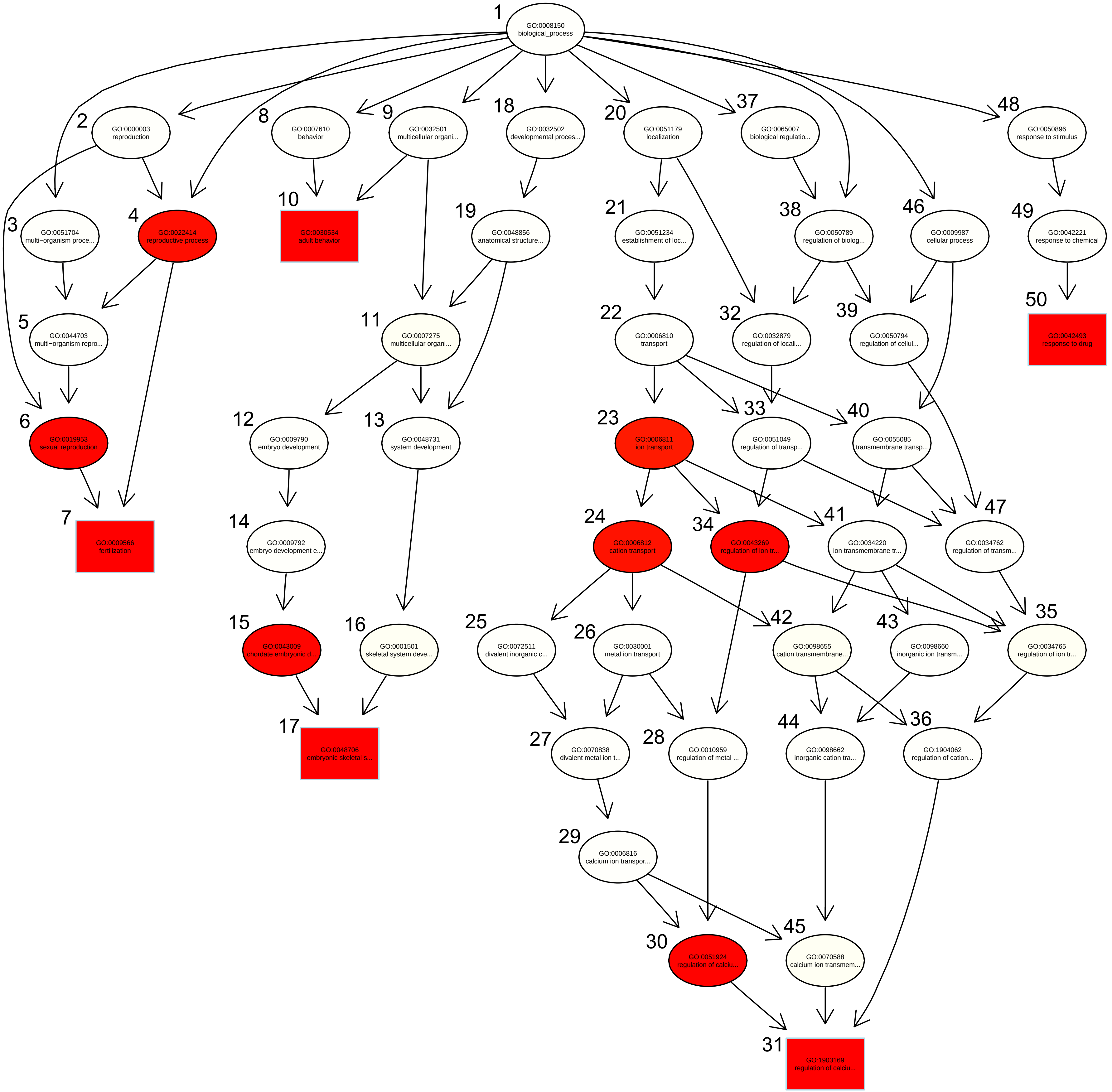

1 GO:0008150 biological_process
2 GO:0000003 reproduction
3 GO:0051704 multi−organism proce...
4 GO:0022414 reproductive process
5 GO:0044703 multi−organism repro...
6 GO:0019953 sexual reproduction
7 GO:0009566 fertilization
8 GO:0007610 behavior
9 GO:0032501 multicellular organi...
10 GO:0030534 adult behavior
11 GO:0007275 multicellular organi...
12 GO:0009790 embryo development
13 GO:0048731 system development
14 GO:0009792 embryo development e...
15 GO:0043009 chordate embryonic d...
16 GO:0001501 skeletal system deve...
17 GO:0048706 embryonic skeletal s...
18 GO:0032502 developmental proces...
19 GO:0048856 anatomical structure...
20 GO:0051179 localization
21 GO:0051234 establishment of loc...
22 GO:0006810 transport
23 GO:0006811 ion transport
24 GO:0006812 cation transport
25 GO:0072511 divalent inorganic c...
26 GO:0030001 metal ion transport
27 GO:0070838 divalent metal ion t...
28 GO:0010959 regulation of metal ...
29 GO:0006816 calcium ion transpor...
30 GO:0051924 regulation of calciu...
31 GO:1903169 regulation of calciu...
32 GO:0032879 regulation of locali...
33 GO:0051049 regulation of transp...
34 GO:0043269 regulation of ion tr...
35 GO:0034765 regulation of ion tr...
36 GO:1904062 regulation of cation...
37 GO:0065007 biological regulatio...
38 GO:0050789 regulation of biolog...
39 GO:0050794 regulation of cellul...
40 GO:0055085 transmembrane transp...
41 GO:0034220 ion transmembrane tr...
42 GO:0098655 cation transmembrane...
43 GO:0098660 inorganic ion transm...
44 GO:0098662 inorganic cation tra...
45 GO:0070588 calcium ion transmem...
46 GO:0009987 cellular process
47 GO:0034762 regulation of transm...
48 GO:0050896 response to stimulus
49 GO:0042221 response to chemical
50 GO:0042493 response to drug

Supplementary Figure S2. Molecular pathway analysis of RHDV2-infected liver transcriptome samples. The subgraph is induced by the top 5 gene ontology (GO) components (depicted in red squares) identified by the weight01 algorithm for scoring gene ontology components (Alexa et al., 2006). White and red ovals depict gene ontology components that contribute to a pathway. Shapes colored red indicate the most significant components, whereas those colored white indicate less significant components. All the components are numbered and a description for each of the components can be found in **Supplementary Table S2** (see below). TopGO R package was used to build the network (Alexa and Rahnenfuhrer, 2020).

References

Alexa, A., and Rahnenfuhrer, J. (2020). topGO: Enrichment Analysis for Gene Ontology.

Alexa, A., Rahnenführer, J., and Lengauer, T. (2006). Improved scoring of functional groups from gene expression data by decorrelating GO graph structure. Bioinformatics 22, 1600–1607. doi:10.1093/bioinformatics/btl140.

Supplementary Table S2

Gene ontology (GO) components from Supplementary Figure S2

#	GO ID	GO component description
1	GO:0008150	Biological process
2	GO:0000003	Reproduction
3	GO:0051704	Multi-organism process
4	GO:0022414	Reproductive process
5	GO:0044703	Multi-organism reproductive process
6	GO:0019953	Sexual reproduction
7	GO:0009566	Fertilization
8	GO:0007610	Behaviour
9	GO:0032501	Multicellular organismal process
10	GO:0030534	Adult behaviour
11	GO:0007275	Multicellular organism development
12	GO:0009790	Embryo development
13	GO:0048731	System development
14	GO:0009792	Embryo development ending in birth or egg hatching
15	GO:0043009	Chordate embryonic development
16	GO:0001501	Skeletal system development
17	GO:0048706	Embryonic skeletal system development
18	GO:0032502	Developmental process
19	GO:0048856	Anatomical structure development
20	GO:0051179	Localization
21	GO:0051234	Establishment of localization
22	GO:0006810	Transport
23	GO:0006811	Ion transport
24	GO:0006812	Cation transport
25	GO:0072511	Divalent inorganic cation transmembrane transport
26	GO:0030001	Metal ion transport
27	GO:0070838	Divalent metal ion transport
28	GO:0010959	Regulation of metal ion transport
29	GO:0006816	Calcium ion transport
30	GO:0051924	Regulation of calcium ion transport
31	GO:1903169	Regulation of calcium ion transmembrane transport
32	GO:0032879	Regulation of localization
33	GO:0051049	Regulation of transport
34	GO:0043269	Regulation of ion transport
35	GO:0034765	Regulation of ion transmembrane transport
36	GO:1904062	Regulation of cation transmembrane transport
37	GO:0065007	Biological regulation
38	GO:0050789	Regulation of biological process
39	GO:0050794	Regulation of cellular process

40	GO:0055085	Transmembrane transport
41	GO:0034220	Ion transmembrane transport
42	GO:0098655	Cation transmembrane transport
43	GO:0098660	Inorganic ion transmembrane transport
44	GO:0098662	Inorganic cation transmembrane transport
45	GO:0070588	Calcium ion transmembrane transport
46	GO:0009987	Cellular process
47	GO:0034762	Regulation of transmembrane transport
48	GO:0050896	Response to stimulus
49	GO:0042221	Response to chemical
50	GO:0042493	Response to xenobiotic stimulus
