# Supplementary material for: Lagovirus Non-structural Protein p23: A Putative Viroporin That Interacts With Heat Shock Proteins and Uses a Disulfide Bond for Dimerization
Source: Front Microbiol. 2022 Jul 7;13:923256. doi: 10.3389/fmicb.2022.923256 (PMC9340658; doi:10.3389/fmicb.2022.923256)
Supplement: Supplementary file 7 [file Data_Sheet_5.PDF]

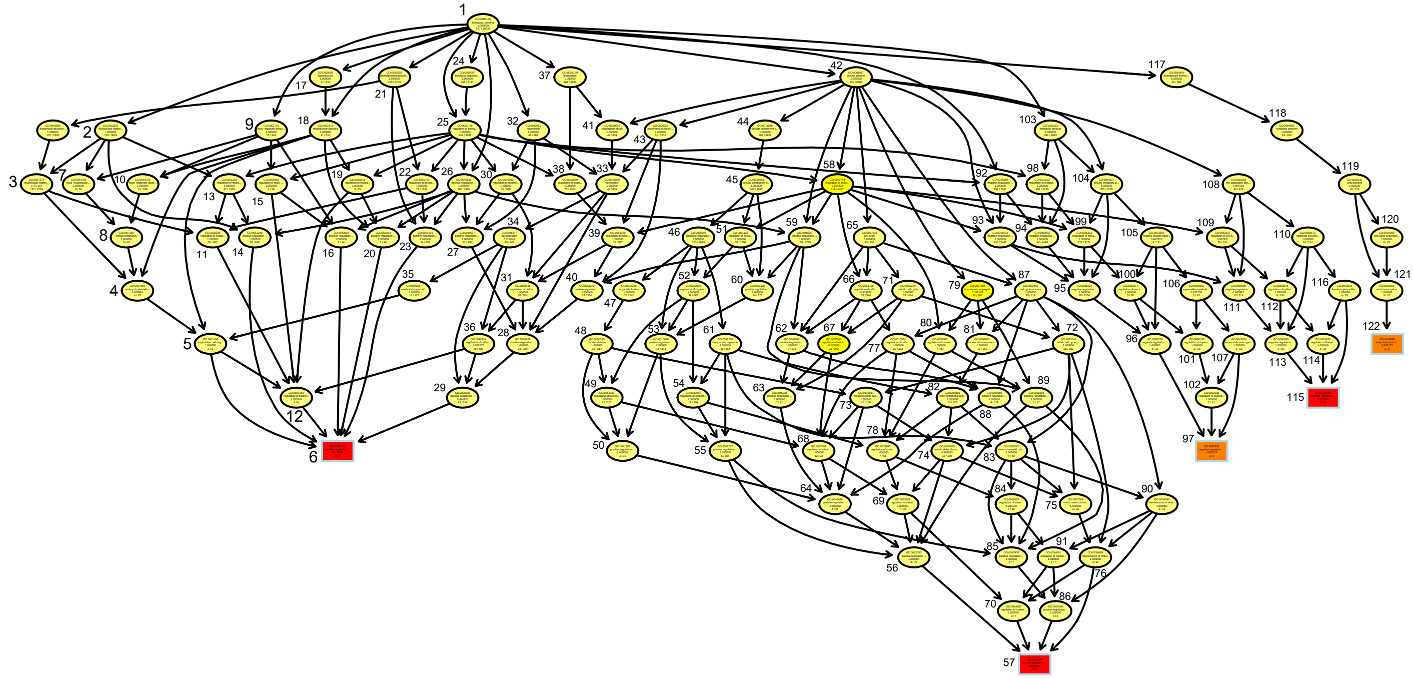
1
2
3
4
5
6
7
8
9
10
11
12
13
14
15
16
17
18
19
20
21
22
23
24
25
26
27
28
29
30
31
32
33
34
35
36
37
38
39
40
41
42
43
44
45
46
47
48
49
50
51
52
53
54
55
56
57
58
59
60
61
62
63
64
65
66
67
68
69
70
71
72
73
74
75
76
77
78
79
80
81
82
83
84
85
86
87
88
89
90
91
92
93
94
95
96
97
98
99
100
101
102
103
104
105
106
107
108
109
110
111
112
113
114
115
116
117
118
119
120
121
122

Supplementary Figure S3. Molecular pathway analysis of RHDV2-infected liver samples (transcriptome). The network subgraph is induced by the top 5 gene ontology (GO) components (depicted in squares) identified by the weight01 algorithm with Fisher's exact test (Alexa et al., 2006). Box colors represent the relative significance of GO components, with yellow, orange, and red boxes and ovals indicating least and most significant GO terms, respectively. All the components are numbered and the description for each of the components can be found in **Supplementary Table S3** (see below). TopGO R package was used to build the network (Alexa and Rahnenfuhrer, 2020).

References

Alexa, A., and Rahnenfuhrer, J. (2020). topGO: Enrichment Analysis for Gene Ontology.

Alexa, A., Rahnenführer, J., and Lengauer, T. (2006). Improved scoring of functional groups from gene expression data by decorrelating GO graph structure. Bioinformatics 22, 1600–1607. doi:10.1093/bioinformatics/btl140.

Supplementary Table S3

Gene ontology (GO) components from Supplementary Figure S3

#	GO ID	GO component description
1	GO:0008150	Biological process
2	GO:0032501	Multicellular organismal process
3	GO:0007275	Multicellular organism development
4	GO:0007566	Embryo implantation
5	GO:0061450	Trophoblast cell migration
6	GO:1901165	Positive regulation of trophoblast cell migration
7	GO:0044706	Multi-multicellular organism process
8	GO:0007565	Female pregnancy
9	GO:0051704	Multi-organism process
10	GO:0044703	Multi-organism reproductive process
11	GO:2000026	Regulation of multicellular organismal development
12	GO:1901163	Regulation of trophoblast cell migration
13	GO:0051239	Regulation of multicellular organismal process
14	GO:0051240	Positive regulation of multicellular organismal process
15	GO:0043900	Obsolete regulation of multi-organism process
16	GO:0043902	Obsolete positive regulation of multi-organism process
17	GO:0000003	Reproduction
18	GO:0022414	Reproductive process
19	GO:2000241	Regulation of reproductive process
20	GO:2000243	Positive regulation of reproductive process
21	GO:0032502	Developmental process
22	GO:0050793	Regulation of developmental process
23	GO:0051094	Positive regulation of developmental process
24	GO:0065007	Biological regulation
25	GO:0050789	Regulation of biological process
26	GO:0048518	Positive regulation of biological process
27	GO:0040017	Positive regulation of locomotion
28	GO:2000147	Positive regulation of cell motility
29	GO:0030335	Positive regulation of cell migration
30	GO:0040012	Regulation of locomotion
31	GO:2000145	Regulation of cell motility
32	GO:0040011	Locomotion
33	GO:0048870	Cell motility
34	GO:0016477	Cell migration
35	GO:0001667	Ameboidal-type cell migration
36	GO:0030334	Regulation of cell migration
37	GO:0051179	Localization
38	GO:0032879	Regulation of localization
39	GO:0051270	Regulation of cellular component movement
40	GO:0051272	Positive regulation of cellular component movement
41	GO:0051674	Localization of cell
42	GO:0009987	Cellular process

43	GO:0006928	Movement of cell or subcellular component
44	GO:0071840	Cellular component organization or biogenesis
45	GO:0016043	Cellular component organization
46	GO:0006996	Organelle organization
47	GO:0048285	Organelle fission
48	GO:0000280	Nuclear division
49	GO:0051783	Regulation of nuclear division
50	GO:0051785	Positive regulation of nuclear division
51	GO:0051128	Regulation of cellular component organization
52	GO:0033043	Regulation of organelle organization
53	GO:0010638	Positive regulation of organelle organization
54	GO:0033044	Regulation of chromosome organization
55	GO:2001252	Positive regulation of chromosome organization
56	GO:0062033	Positive regulation of mitotic sister chromatid segregation
57	GO:0034184	Positive regulation of maintenance of mitotic sister chromatid cohesion
58	GO:0050794	Regulation of cellular process
59	GO:0048522	Positive regulation of cellular process
60	GO:0051130	Positive regulation of cellular component organization
61	GO:0051276	Chromosome organization
62	GO:0045787	Positive regulation of cell cycle
63	GO:0045931	Positive regulation of mitotic cell cycle
64	GO:0045840	Positive regulation of mitotic nuclear division
65	GO:0007049	Cell cycle
66	GO:0051726	Regulation of cell cycle
67	GO:0007346	Regulation of mitotic cell cycle
68	GO:0007088	Regulation of mitotic nuclear division
69	GO:0033047	Regulation of mitotic sister chromatid segregation
70	GO:0034182	Regulation of maintenance of mitotic sister chromatid cohesion
71	GO:0000278	Mitotic cell cycle
72	GO:1903047	Mitotic cell cycle process
73	GO:0140014	Mitotic nuclear division
74	GO:0000070	Mitotic sister chromatid segregation
75	GO:0007064	Mitotic sister chromatid cohesion
76	GO:0034088	Maintenance of mitotic sister chromatid cohesion
77	GO:0010564	Regulation of cell cycle process
78	GO:0033045	Regulation of sister chromatid segregation
79	GO:0007059	Chromosome segregation
80	GO:0051983	Regulation of chromosome segregation
81	GO:0098813	Nuclear chromosome segregation
82	GO:0000819	Sister chromatid segregation
83	GO:0007062	Sister chromatid cohesion
84	GO:0007063	Regulation of sister chromatid cohesion
85	GO:0045876	Positive regulation of sister chromatid cohesion
86	GO:0034093	Positive regulation of maintenance of sister chromatid cohesion
87	GO:0022402	Cell cycle process
88	GO:0090068	Positive regulation of cell cycle process
89	GO:0051984	Positive regulation of chromosome segregation
90	GO:0034086	Maintenance of sister chromatid cohesion

91	GO:0034091	Regulation of maintenance of sister chromatid cohesion
92	GO:0048519	Negative regulation of biological process
93	GO:0048523	Negative regulation of cellular process
94	GO:0009892	Negative regulation of metabolic process
95	GO:0031324	Negative regulation of cellular metabolic process
96	GO:2000378	Negative regulation of reactive oxygen species metabolic process
97	GO:0032929	Negative regulation of superoxide anion generation
98	GO:0019222	Regulation of metabolic process
99	GO:0031323	Regulation of cellular metabolic process
100	GO:2000377	Regulation of reactive oxygen species metabolic process
101	GO:0090322	Regulation of superoxide metabolic process
102	GO:0032928	Regulation of superoxide anion generation
103	GO:0008152	Metabolic process
104	GO:0044237	Cellular metabolic process
105	GO:0072593	Reactive oxygen species metabolic process
106	GO:0006801	Superoxide metabolic process
107	GO:0042554	Superoxide anion generation
108	GO:0008283	Cell population proliferation
109	GO:0042127	Regulation of cell population proliferation
110	GO:0050673	Epithelial cell proliferation
111	GO:0008285	Negative regulation of cell population proliferation
112	GO:0050678	Regulation of epithelial cell proliferation
113	GO:0050680	Negative regulation of epithelial cell proliferation
114	GO:0010837	Regulation of keratinocyte proliferation
115	GO:0010839	Negative regulation of keratinocyte proliferation
116	GO:0043616	Keratinocyte proliferation
117	GO:0044419	Biological process involved in interspecies interaction between organisms
118	GO:0044403	Biological process involved in symbiotic interaction
119	GO:0016032	Viral process
120	GO:0019080	Viral gene expression
121	GO:0019083	Viral transcription
122	GO:0019085	Early viral transcription
